# Inhibition of EPAC1 Prevents Neuronal Death Mediated by Diesel Exhaust Particles in Ferroptotic Cell Death Conditions

**DOI:** 10.3390/antiox15050566

**Published:** 2026-04-29

**Authors:** Hong Yan, Leshan Zhang, Ana L. Manzano-Covarrubias, Phoeja S. Gadjdjoe, Anja Land, Christina H. J. T. M. van der Veen, Teresa Mitchell-Garcia, Heba A. Fayyaz, Marco Venema, Christoffer Åberg, Marieke van der Hart, Frank Lezoualc’h, Xiaodong Cheng, Amalia M. Dolga, Martina Schmidt

**Affiliations:** 1 Department of Molecular Pharmacology, Groningen Research Institute of Pharmacy (GRIP), Faculty of Science and Engineering, University of Groningen, Antonius Deusinglaan 1, 9713 AV Groningen, The Netherlands; h.yan@em.uni-frankfurt.de (H.Y.); l.z.zhang@rug.nl (L.Z.); a.l.manzano.covarrubias@rug.nl (A.L.M.-C.); phoeja@zuiderparkapotheek.nl (P.S.G.); anja.land@uni-kostanz.de (A.L.); c.h.t.j.mol-van.der.veen@rug.nl (C.H.J.T.M.v.d.V.); teresa.mitchell.garcia@rug.nl (T.M.-G.);; 2 Groningen Research Institute of Asthma and COPD (GRIAC), University Medical Center Groningen (UMCG), University of Groningen, 9713 AV Groningen, The Netherlands; 3 Department of Nanomedicine and Drug Targeting, Groningen Research Institute of Pharmacy, Faculty of Science and Engineering, University of Groningen, Antonius Deusinglaan 1, 9713 AV Groningen, The Netherlands; h.fayyaz@rug.nl; 4 Department of Pharmaceutical Analysis, Groningen Research Institute of Pharmacy, Faculty of Science and Engineering, University of Groningen, Antonius Deusinglaan 1, 9713 AV Groningen, The Netherlands; christoffer.aberg@rug.nl; 5 Analytical Biochemistry, Groningen Research Institute of Pharmacy (GRIP), Faculty of Science and Engineering, University of Groningen, Antonius Deusinglaan 1, 9713 AV Groningen, The Netherlands; 6 Quantall B.V., L.J. Zielstraweg 1, 9713 GX Groningen, The Netherlands; 7 Université de Toulouse, Inserm, Institut des Maladies Métaboliques et Cardiovasculaires, I2MC, 31432 Toulouse, France; frank.lezoualch@inserm.fr; 8 Department of Integrative Biology & Pharmacology, Texas Therapeutics Institute, University of Texas Health Science Center at Houston, Houston, TX 7000, USA; xiaodong.cheng@uth.tmc.edu

**Keywords:** diesel exhaust particle, ferroptosis, EPAC, soluble AC, cAMP, oxidative stress

## Abstract

Air pollution is a growing hazard to global health. Epidemiological studies have reported a potential role of air pollutant exposure in the development or aggravation of neurodegenerative diseases. However, the underlying mechanisms are ill-defined. Ferroptosis is an iron- and reactive oxygen species (ROS)-dependent form of cell death that drives neuronal loss in neurodegenerative diseases. Our previous studies reported the involvement of adenosine 3′,5′-cyclic monophosphate (cAMP) and EPAC (exchange protein directly activated by cAMP) in ferroptotic cell death. Here, we investigated the effects of diesel exhaust particles (DEP) in mouse hippocampal (HT22) neuronal cells. Our data showed that toxicity induced by RSL3 (50–75 nM), a ferroptosis inducer, was significantly increased by the addition of DEP (100 μg/mL). Pharmacological inhibition of EPAC1 (CE3F4 30 μM or AM-001 30 μM) and soluble adenylyl cyclase (sAC; TDI-10229 1 μM or TDI-11861 0.1 μM) prevented enhanced ferroptotic HT22 cell death caused by DEP, while pharmacological modulation of EPAC2, protein kinase A (PKA), phosphodiesterases (PDEs), or transmembrane AC did not. DEP in combination with RSL3 exposure increased intracellular calcium levels and induced lysosomal de-acidification. Furthermore, inhibition of EPAC1 prevented mitochondrial ROS (MitoSOX) and lipid peroxidation (BODIPY C11 and MDA levels) after DEP and RSL3 co-exposure. Collectively, EPAC1 may serve as a novel target for the treatment or prevention of neurodegenerative diseases accelerated by air pollution.

## 1. Introduction

Exposure to air pollution has been a significant threat to human health. The report “Ambient (outdoor) air pollution” issued by the World Health Organization (WHO) stated that about 4.2 million premature deaths worldwide in 2019 were caused by outdoor air pollution [1]. According to the State of Global Air 2025 report, air pollution accounted for approximately 7.9 million deaths worldwide in 2023, with ambient PM2.5 as the dominant contributor, and 6.8 million (86%) of these attributable to noncommunicable diseases [2]. It has been estimated that only 1% of the world’s population lives in places that meet the WHO air quality guidelines [3].

Particulate matter (PM) in air pollution is considered to have greater adverse health effects compared to other pollutants [4], being associated with various cardiovascular, respiratory, and neurological diseases [5]. DEP, a major component of PM, induces oxidative stress by generating reactive oxygen species (ROS) via its carbon-based core and associated polycyclic aromatic hydrocarbons (PAHs) [6]. Fine particles in PM can translocate to the brain, exacerbating neuronal oxidative stress and contributing to neurodegenerative diseases [7]. Magnetic particles (Fe_3_O_4_), with a diameter of less than 150 nanometers in air pollutants, have been found in the frontal cortex of the human brain [8]. Long-term exposure to air pollution can significantly accelerate the development of cognitive alterations by a variety of cellular, molecular, oxidative stress, and neuroinflammation pathways [9].

In addition, some studies have reported that exposure to PM exacerbates brain injury in the presence of underlying diseases such as Alzheimer’s disease (AD) and Parkinson’s disease (PD) [10]. AD and PD mouse models exposed to PM showed increased amyloid beta plaque accumulation, primarily attributed to ROS-induced protein misfolding and aggregation [11] and alpha-synuclein aggregation [12]. Ferroptosis, a type of oxidative cell death that occurs in neurodegenerative diseases [13], has emerged as a critical mechanism underlying the neurotoxic effects of PM exposure [14]. PM2.5 induced ferroptosis in SH-SY5Y human neuroblastoma cells with increased iron, MDA, and lipid ROS levels [15]. However, it is unclear whether ferroptosis is related to PM-increased neurodegeneration.

The second messenger cyclic adenosine 3′,5′-monophosphate (cAMP) is tightly controlled by phosphodiesterases (PDEs) and plays an important role in cellular responses to many extracellular stimuli, which may be involved in pollutant-mediated oxidative stress and mitochondrial dysfunction [5]. Potential physiological effects of cAMP are diverse, including proteins sensitive to cAMP, such as exchange protein directly activated by cAMP (EPAC) and protein kinase A (PKA). The effects of PKA on ferroptosis may vary depending on the specific context, as PKA can exhibit both pro- and anti-ferroptotic actions [16]. EPAC has been identified as a potential therapeutic target in the central nervous system (CNS) [17,18]. EPAC1 expression is upregulated while EPAC2 expression level is downregulated in the hippocampal and frontal cortex of AD patients [19]. There is an upregulation of EPAC1 protein levels in inflamed neurons of rats [20] and a downregulation of EPAC2 in hippocampal postmortem samples of AD patients [21,22]. EPAC signaling is reported to be involved in cell death in the CNS, such as apoptosis in mouse cortical neurons [23] and autophagy in PC-12 cells [24]. We also reported that pharmacological inhibition of EPAC1 in HT22 cells prevented ferroptosis by decreasing mitochondrial superoxide levels [25].

Cyclic AMP is generated from ATP by members of the adenylyl cyclase (AC) family [26]. There are nine transmembrane ACs (TmACs) encoded by AC1 to nine genes and one soluble AC enzyme (sAC) encoded by the ADCY10 gene. sAC, stimulated by bicarbonate (HCO_3_^−^) and calcium ion (Ca^2+^), is structurally and biochemically distinct from G protein-responsive TmACs, which allows localization independent of membrane compartments [27]. EPAC1 also plays a significant role in modulating Ca^2+^ signaling pathways [28], which may contribute to the activation of sAC. However, the role of sAC, cAMP, and EPAC1 in cell death in the CNS is less well known; to the best of our knowledge, no study has linked them to the determination of the ferroptosis sensitivity caused by PM.

Nanometer-sized DEP is used in the literature as a model of ultrafine PM [29], as this is the approximate size of PM translocated to the brain and the PM fraction with the neurotoxic effects [30]. Therefore, we used DEP to investigate whether and how it enhances ferroptosis sensitivity by affecting sAC/EPAC in ferroptotic HT22 cells induced by RSL3.

## 2. Materials and Methods

### 2.1. Particle Preparation

Standard reference material (SRM) 2975 DEP (Industrial Forklift, #1333-86-4), commonly used as a standardized diesel exhaust particle sample, was purchased from the National Institute of Standards and Technology (NIST; Gaithersburg, MD, USA). DEP particles have a mean diameter of 37.51 ± 1.56 nm [31], primarily consist of carbon black, polycyclic aromatic hydrocarbons (PAHs), and nitro-PAHs, as per the NIST certificate of analysis [32]. No specific iron or calcium content is reported for this standard.

DEP working suspensions were prepared according to Park et al. [33]. Briefly, 5 mg of DEP was suspended in 2 mL phosphate-buffered solution (PBS) at a final concentration of 2.5 mg/mL. The suspension was then subjected to bath sonication for 30 min and probe sonication for 30 seconds. To prevent sedimentation of DEP, all suspensions were vortexed vigorously for 1 min immediately before addition to the cells; 0.1% (*v*/*v*) Tween 80 (#9005-65-6; Sigma-Aldrich, St. Louis, MO, USA) was included as a dispersant, as previously described [34,35]. The control group used PBS without particles and followed the same steps. No significant cytotoxicity was observed with the Tween 80-containing vehicle control (Figure S5). All substances were purchased from Sigma-Aldrich, unless otherwise indicated.

### 2.2. Cell Culture and Treatment

HT22 cells were grown on tissue culture flasks and were maintained at 37 °C, 5% CO_2_ in Dulbecco’s modified Eagle Medium glucose (#42430025; Gibco, Thermo Fisher Scientific, Waltham, MA, USA) supplemented with 10% fetal bovine serum (#SV3016.03; Cytiva HyClone, Marlborough, MA, USA), 2% penicillin–streptomycin (#15070063; 5 × 10^3^ U/mL; Gibco, Thermo Fisher Scientific, Waltham, MA, USA), and 1% sodium pyruvate (#11360070; 100 mM; Gibco, Thermo Fisher Scientific, Waltham, MA, USA) [36]. Cells regularly underwent testing for mycoplasma contamination, and only mycoplasma-free cells were used in the study. To ensure consistency across cell passage numbers (270–350), we employed cell viability assays with various stressors to confirm comparable cellular stress response kinetics, as described by [37].

Upon 80% confluence, HT22 cells were treated with different concentrations (3, 10, 30, and 100 μg/mL) of DEP suspended in the culture medium. The concentrations of DEP used in this study were selected based on extrapolations from in vivo exposure levels (~10–100 μg/m^3^) reported by Li et al. [38] and previous toxicology studies [15,39,40,41]. Immediately before treatment, all the suspensions were vortexed for 1 min to obtain a proper particle dispersion. Cells were treated with DEP for 24 h; in parallel, control cells were treated with culture medium supplemented with 0.1% (*v*/*v*) Tween 80 in sonicated PBS.

RSL3 (#S8155; Selleckchem, Houston, TX, USA) concentrations (25–500 nM, with 50 and 75 nM used in combination with DEP) were chosen based on their ability to induce ferroptosis without profound cytotoxicity alone (Figure S1A). Inhibitors and activators were selected based on their established specificity and effective concentrations in prior studies and preliminary dose–response experiments (Figure S1). A total of 10 μM Pan-caspase inhibitor QVD (#1135695-98-5; MP Biomedicine, Solon, OH, USA) [42], 5 μM ferroptosis inhibitor ferrostatin-1 (Fer-1; #SML0583; Scientific Laboratory Supplies, Rathcoole, Co. Dublin, Ireland) [36], EPAC1 inhibitors, 3–100 μM CE3F4 and 30 μM AM-001 (from Dr. Frank Lezoualc) [43,44], 100 μM PKA inhibitor Rp-8CPT-cAMPs (#129735-01-9; Biolog Life Science Institute, Bremen, Germany) [45], 10 μM EPAC2 inhibitor ESI-05 (from Dr. Xiaodong Cheng) [46], 10 μM EPAC2 activator Sp-8-BnT-cAMPS (S220; #B 046-05; Biolog Life Science Institute, Bremen, Germany) [46], 30 μM PDE4 inhibitor rolipram (#61413-54-5; Tocris Bioscience, Bristol, UK) [47], 30 μM PDE3 inhibitor cilostamide (#68550-75-4; Tocris Bioscience, Bristol, UK) [47], 1 μM sAC inhibitor TDI-10229 and 0.1 μM TDI-11861 (from Dr. Lonny Levin and Dr. Jochen Buck), 30 μM TmAC activator Forskolin (#66575-29-9; Tocris Bioscience, Bristol, UK), and 10 μM TmAC inhibitor 2′,5′-dideoxyadenosine (dd-Ado; #6698-26-6; Merck, Darmstadt, Germany) were used. Exposure time for Ca^2+^, mitochondrial ROS (mtROS), and lipid peroxidation experiments was approximately 6 h, a time point that precedes significant death induction. All other co-treatment exposure times were 17 h.

### 2.3. Cell Metabolic Assay

Cell metabolic activity was measured using the 3-[4,5-dimethylthiazol-2-yl]-2,5-diphenyl-2H-tetrazolium bromide (MTT; #5655; Sigma-Aldrich, St. Louis, MO, USA) assay [36]. HT22 cells were seeded onto 96-well plates at a density of 9000 cells/well. After the desired treatment, cells were washed with PBS. MTT stock solution (5 mg/mL) was added to each plate to a final concentration of 0.5 mg/mL, and cells were incubated for 1 h at 37 °C. After removing the MTT solution, plates were incubated at −20 °C for 1 h. Next, 70 μL dimethyl sulfoxide (DMSO) (#D8418, Lot#SHBH9942; Sigma-Aldrich, St. Louis, MO, USA) was added to each well, and plates were shaken at 150 rpm for 40 min. The absorbance was measured at a wavelength of 570 and 630 nm to correct for non-specific absorbance using a Synergy H1 Multi-Mode reader (Biotek, Winooski, VT, USA).

### 2.4. Double Staining with Hoechst 33258 and PI

HT22 cells were seeded onto 8-well ibidi plates at a density of 20,000 cells/well. After the indicated treatment, cells were double-stained with 1 μg/mL Hoechst 33258 (#H3570; Invitrogen, Thermo Fisher Scientific, Waltham, MA, USA) and 3 μM propidium iodide (PI; #V13242; Fisher Scientific, Landsmeer, The Netherlands) at 37 °C for 10 min in the dark. Images were acquired on a Nikon Inverted Research Fluorescence Microscope ECLIPSE Ti2-E at a constant exposure of 100 ms. At least 10 images of each well were captured at magnifications of 40× (excitation/emission: 350/461 nm for Hoechst and 535/617 nm for PI). Percentages of cell death were calculated by determining the ratio of PI-stained cells to Hoechst-stained cells.

### 2.5. cAMP Measurements

cAMP content was determined using liquid chromatography with tandem mass spectrometry (LC-MS/MS) as previously described [48,49]. HT22 cells were seeded onto 6-well plates at a density of 250,000 cells/well. After treatments, cells were scraped into ice-cold water, lysed by ultrasound and three freeze–thaw cycles, and centrifuged at 8000× *g* for 10 min. Supernatants were analyzed on an integrated HPLC system (prominence series; Shimadzu, Kyoto, Japan) using an API-4000 triple quad mass spectrometer (AB Sciex, Marlborough, MA, USA). Ultrapure water with 0.1% formaldehyde (FA) was used as mobile phase A (MPA), and acetonitrile containing 0.1% FA was used as mobile phase B (MPB). Separations were performed on a 2.1 × 150 mm, 3.0 µm, 100 A pore C18 column (Atlantis T3, USA) with a linear gradient from 0% to 65% of MPB. The injection volume was 5 µL, and the mobile phase flow rate was 0.3 mL per min. The column temperature was held constant at 40 °C, while the autosampler was set to 6 °C. The mass spectrometer was operated in MRM positive ionization mode. The *m*/*z* transitions—330.1/136.2 transition for cAMP and 335.1/136.2 transition for the internal standard, ^13^C_5_-cAMP (Toronto Research Chemicals, North York, ON, Canada), were used for quantification, as previously described [49]. Results were analyzed using Analyst 1.6.2. Software (AB Sciex, Marlborough, MA, USA). The calibration curve was prepared in a matched matrix with a lower limit of quantification of 0.5 nM, and quality control samples were used throughout the analysis to assess system suitability.

### 2.6. Intracellular Ca^2+^ Level Detection

HT22 cells were seeded onto 8-well ibidi plates at a density of 20,000 cells/well. After treatment, cells were rinsed twice with Hanks’ balanced salt solution (HBSS) (#14065-056; Gibco, Thermo Fisher Scientific, Waltham, MA, USA), and then incubated with 3 μM Fluo-4 AM (# F14201; Invitrogen, Thermo Fisher Scientific, Waltham, MA, USA) and 1 μg/mL Hoechst 33258 at 37 °C for 10 min in the dark [50]. The plate was rinsed twice with HBSS and immediately analyzed using a Nikon Inverted Research Fluorescence Microscope ECLIPSE Ti2-E at a constant exposure of 100 ms (excitation/emission: 494/516 for Fluo-4 AM). At least 10 images of each well were captured at magnifications of 20×. The fluorescence intensity of Fluo-4 AM was quantified using the Fiji/ImageJ software (version 1.54p; National Institutes of Health, Bethesda, MD, USA).

### 2.7. mtROS Measurement

HT22 cells were seeded onto 8-well ibidi µ-Slide plates at 20,000 cells/well or in 96-well black-walled plates at 9000 cells/well. After treatment, 1.25 μM MitoSOX red reagent (#36008; Fisher Scientific, Landsmeer, The Netherlands) and 1 μg/mL Hoechst 33258 (#H3570; Invitrogen, Thermo Fisher Scientific, Waltham, MA, USA) were added to cells and incubated at 37 °C for 30 min in the dark. Plates were rinsed twice with HBSS.

Imaging was performed on a Nikon ECLIPSE Ti2-E Microscope at a constant exposure of 100 ms. At least 10 images of each well were captured at magnifications of 20×. The fluorescence intensity of MitoSOX red was quantified using the Fiji/ImageJ software (version 1.54p; National Institutes of Health, Bethesda, MD, USA). Plate-reader quantification was carried out on a Synergy H1 Multi-Mode reader at excitation/emission 510/580 nm and was performed as described previously [51,52].

### 2.8. Lipid Peroxidation Assay

Lipid ROS levels were measured as described previously [53]. HT22 cells were seeded onto 8-well ibidi plates at a density of 20,000 cells/well. After treatment, 1.5 μM BODIPY 581/591 C11 (#D3861; Fisher Scientific, Landsmeer, The Netherlands) and 1 μg/mL Hoechst 33258 were added to cells, and incubated at 37 °C for 30 min in the dark. Fluorescence was measured at Ex/Em = 581/591 nm (reduced state) and 488/510 nm (oxidation) using a Nikon ECLIPSE Ti2-E Microscope. At least 10 images of each well were captured at magnifications of 20×. The fluorescence intensity of BODIPY 581/591 C11 (red/green) was quantified using the Fiji/ImageJ software (version 1.54p; National Institutes of Health, Bethesda, MD, USA).

### 2.9. Thiobarbituric Acid Reactive Substances (TBARS)

Malondialdehyde (MDA), as a marker of lipid peroxidation, was measured by the TBARS method [54]. HT22 cells were seeded onto 6-well plates at a density of 250,000 cells/well. After treatments, cells were scraped into ice-cold water, lysed by ultrasound and three freeze–thaw cycles, mixed with 10% trichloroacetic acid (15 min on ice), and centrifuged at 5000× *g* for 15 min. The supernatant was reacted with 0.75% thiobarbituric acid (95 °C for 15 min), cooled, and centrifuged at 3500 r/min for 10 min. Fluorescence of the supernatant was measured at excitation/emission 530/590 nm on a Synergy H1 reader (optimized to reduce background). The data were normalized by protein concentrations determined by the BCA protein assay.

### 2.10. Immunofluorescence

HT22 cells were seeded onto removable 8-well ibidi plates at a density of 20,000 cells/well. After treatment, cells were stained with 1 μM MitoTracker™ Deep Red FM (#22426; Fisher Scientific, Landsmeer, The Netherlands) at 37 °C for 30 min in the dark, rinsed twice with warm PBS, and fixed with 4% paraformaldehyde solution at room temperature for 15 min. After blocking with 1% BSA in PBS for 1 h, cells were incubated overnight at 4 °C with EPAC Antibody (A-5): sc-28366 (1:500, #F1616; Santa Cruz Biotechnology, Dallas, TX, USA) and sAC antibody (R21, 1:100, from Dr. Lonny Levin and Dr. Jochen Buck). Secondary labeling was performed with Alexa Fluor 488-conjugated donkey anti-mouse IgG (H + L) (#A-21202; Invitrogen, Thermo Fisher Scientific, Waltham, MA, USA) for 1 h. Then the plates were rinsed 3 times with PBS, and the chamber was removed. Coverslips were mounted onto slides using an antifade mounting medium with DAPI (#ab104139; Abcam, Cambridge, UK). The fluorescence was observed using a Nikon ECLIPSE Ti2-E microscope. Images of each well were captured at a magnification of 60×, and co-localization was evaluated using the Fiji/ImageJ software (version 1.54p; National Institutes of Health, Bethesda, MD, USA).

### 2.11. Western Blotting

Cells were lysed in RIPA buffer (65 mM Tris, 155 mM NaCl, 1% Igepal CA-630, 0.25% sodium deoxycholate, 1 mM EDTA, pH 7.4) supplemented with protease inhibitors (1 mM Na3VO4, 1 mM NaF, 10 μg/mL leupetin, 10 μg/mL pepstatin A, 10 μg/mL aprotinin). Protein concentration was determined by the BCA assay. Equal amounts of protein were separated on 10% SDS-polyacrylamide gel by electrophoresis and were transferred to nitrocellulose membranes. After blocking the membranes with Roti-Block (Carl Roth, Karlsruhe, Germany), proteins were probed with primary antibodies for β-actin (1:3000, #B2724; Santa Cruz Biotechnology, Dallas, TX, USA), for phospho-PKA substrates (1:1000 dilution, #9624S, Cell Signaling, Danvers, MA, USA) and EPAC1 (5D3) (1:1000, #4155; Cell Signaling Technology, Danvers, MA, USA) at 4 °C. After thorough washing, secondary antibodies (anti-mouse IgG, 1: 5000; Sigma-Aldrich, St. Louis, MO, USA) were used for detection at RT for 2 h. The antigen–antibody complexes were detected using a Western detection ECL-plus kit (PerkinElmer, Waltham, MA, USA).

### 2.12. LysoTracker Staining and Lysosomal Analysis

To determine whether DEP is internalized by HT22 cells, cells were seeded at a density of 30,000 cells/dish in 35 mm glass-bottom MatTek dishes (10 mm microwell, No. 1.5 coverglass) 24 h before treatment. The cells were treated with 10 μg/mL DEP dispersed in DMEM for 24 h (37 °C, 5% CO_2_). Cells were subsequently stained with 0.75 µM LysoTracke Red DND-99 (#L7528; Invitrogen, Thermo Fisher Scientific, Waltham, MA, USA), incubated for 1 h at 37 °C in the dark, washed with pre-warmed PBS, and imaged live using a Nikon Inverted Research Fluorescence Microscope ECLIPSE Ti2-E.

For kinetic imaging, HT22 cells were seeded into 96-well plates at 9000 cells/well. After staining with Lysoview 540 reagent (#70061, Biotium, Fremont, CA, USA) for 30 min, phase-contrast and red fluorescence images were acquired hourly for 24 h at 10× magnification using an Incucyte live-cell imaging system. Red-channel integrated fluorescence intensity was quantified after background exclusion by adaptive segmentation thresholding and normalized to the pre-treatment time point (T0).

For flow cytometric analysis, cells were seeded into 24-well plates at 35,000 cells/well, washed once with cell culture medium with 10% FBS and twice with PBS, and harvested by incubation with trypsin for 5 min at 37 °C and transferred to flow cytometry tubes. Next, cells were centrifuged for 5 min at 300× *g*. The supernatant was discarded, and cell pellets were resuspended in 200 µL cell culture medium with 10% FBS containing 50 nM LysoTracker™ Green DND-26 (#L7526; Invitrogen, Thermo Fisher Scientific, Waltham, MA, USA) and incubated for 20 min at 37 °C in the dark. After staining, cells were pelleted as described above, resuspended in PBS, and analyzed on a CytoFLEX S flow cytometer (Beckman Coulter, Brea, CA, USA). Cells were gated in the forward-side, double-scatter plot to exclude cell debris and cell doublets, and 15,000 cells were acquired for each sample.

### 2.13. Statistical Analysis

Data were expressed as mean ± SD for all experiments. Normality of data distributions was assessed using the Shapiro–Wilk test, and homogeneity of variances was evaluated using Levene’s test. When data satisfied the assumptions of normality and equal variance, statistical analysis was performed using an unpaired Student’s *t*-test or ANOVA followed by Tukey’s post hoc test for multiple comparisons. GraphPad Prism software (version 8.0, GraphPad Software Inc., La Jolla, CA, USA) was used to analyze data. Significance was defined as * *p* < 0.05, ** *p* < 0.01, *** *p* < 0.001, **** *p* < 0.0001, or otherwise not significant (ns).

## 3. Results

### 3.1. DEP Sensitizes RSL3-Induced HT22 Ferroptotic Cell Death

**Figure 1:**
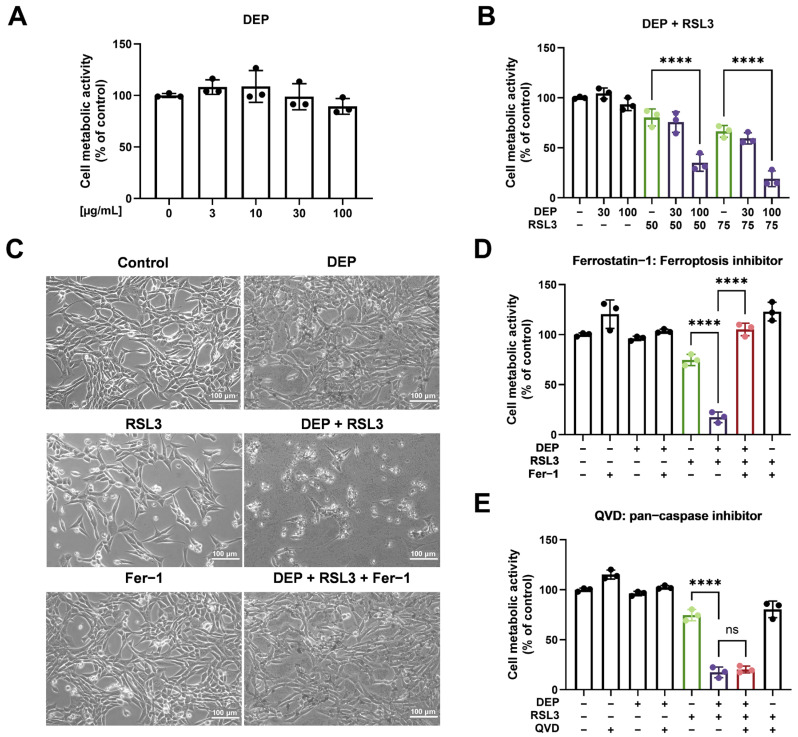
DEP sensitizes HT22 cells to RSL3-induced ferroptotic cell death. (**A**) HT22 cells were treated with DEP at the indicated concentrations for 24 h. (**B**) HT22 cells were co-treated with the indicated concentrations of DEP and RSL3 for 17 h. (**C**) Representative bright-field images of HT22 cells exposed to DEP (100 µg/mL), RSL3 (75 nM), and/or ferrostatin-1 (Fer-1, 5 μM) for 17 h. Scale bar = 100 µm. (**D**) HT22 cells were co-treated with DEP (100 µg/mL) and RSL3 (75 nM) in the absence or presence of ferroptosis inhibitor (Fer-1, 5 μM) for 17 h. (**E**) HT22 cells were co-treated with DEP (100 µg/mL) and RSL3 (75 nM) in the absence or presence of pan-caspase inhibitor (QVD, 10 μM) for 17 h. Cell metabolic activity was assayed by the MTT assay. Data are presented as mean ± SD, each experiment containing 3 technical replicates. One-way ANOVA statistical analysis was used, ns *p* > 0.05, **** *p* < 0.0001. All experiments were independently repeated at least three times.

To determine whether DEP modulates sensitivity to ferroptotic cell death, we treated HT22 cells with increasing concentrations of DEP as previously reported [55,56,57]. Firstly, cells were exposed to DEP in a concentration range of 3, 10, 30, and 100 μg/mL, followed by measurement of their metabolic activity using the MTT assay. As reported in Figure 1A, DEP treatment alone at concentrations lower than 100 μg/mL did not cause any significant change in the cell metabolic activity. Therefore, concentrations up to 100 μg/mL DEP were considered as non-cytotoxic in HT22 cells. Because ferroptosis is one of the mechanisms of neurodegenerative disease, we used RSL3 (25–500 nM) here to trigger ferroptosis. As shown in Figure S1A, treatment with RSL3 inhibited the metabolic activity of cells in a concentration-dependent manner. To determine any potential additional cytotoxic effect of DEP in RSL3-induced ferroptosis, HT22 cells were treated with different combinations of DEP and RSL3 at such concentrations that each compound separately did not profoundly change metabolic activity. Interestingly, we found that 100 μg/mL DEP significantly accelerated the reduction in metabolic activity at low concentrations of RSL3 (50 and 75 nM) compared to the treatment with these compounds alone (Figure 1B). As expected, bright-field images (Figure 1C) showed that single treatment with 100 μg/mL DEP did not affect the cell morphology compared to control HT22 cells, and single treatment with 75 nM RSL3 only had a slight effect on the cell morphology, indicated by some shrinking cells. Consistent with results from the MTT assay, co-treatment significantly altered the cell morphology with large numbers of rounded and detached cells. The ferroptosis inhibitor ferrostatin-1 effectively prevented these morphological changes. Moreover, ferrostatin-1 prevented the inhibition of cell metabolic activity following RSL3 and DEP co-treatment (Figure 1D). In contrast, an inhibitor of apoptosis (QVD) had little impact on cell death mediated by RSL3 and DEP co-treatment (Figure 1E). These results suggested that DEP acts primarily as a ferroptosis sensitizer rather than as an independent ferroptotic trigger under the present experimental conditions. In the following experiments, we applied the combination of 100 μg/mL DEP and 75 nM RSL3 to study the underlying molecular mechanisms.

### 3.2. DEP and RSL3 Induced EPAC1-Dependent HT22 Cell Death

**Figure 2:**
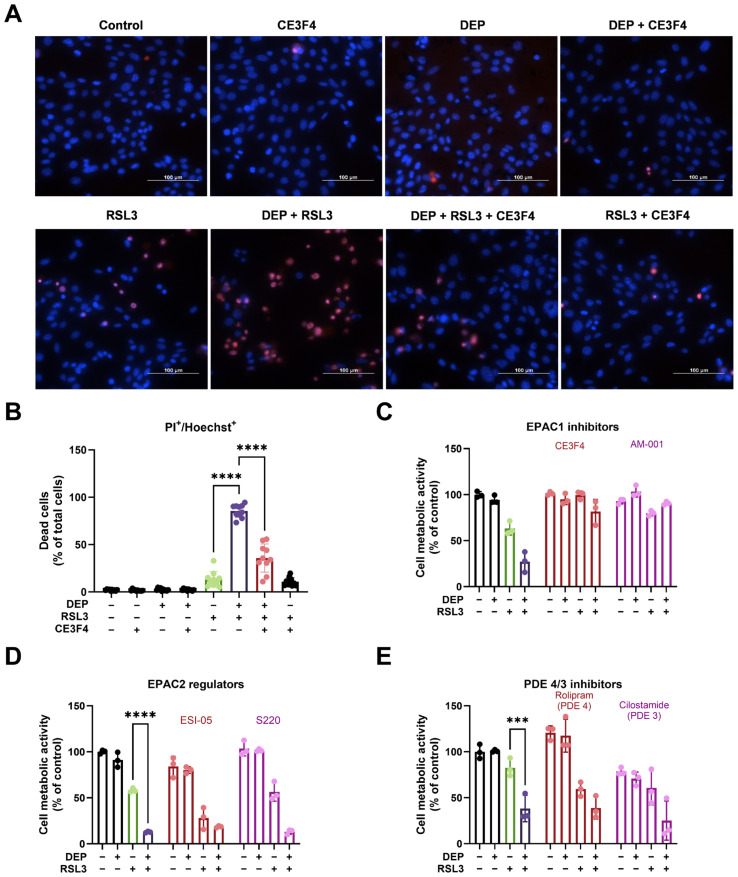
DEP enhances RSL3-induced ferroptosis through EPAC1. (**A**) Representative fluorescence images of PI and Hoechst 33452 staining in HT22 cells after co-treatment with DEP (100 µg/mL) and RSL3 (75 nM) in the absence or presence of EPAC1 inhibitor (CE3F4, 30 µM) for 17 h. Red, PI-positive dead cells; blue: Hoechst-stained nuclei. Scale bar = 100 µm. (**B**) Quantification of the percentage of PI-positive cells relative to total Hoechst-positive cells. Data are presented as mean ± SD, with 10 randomly selected fields analyzed per condition in each experiment. (**C**) HT22 cells were co-treated with DEP (100 µg/mL) and RSL3 (75 nM) in the absence or presence of EPAC1 inhibitors (CE3F4 and AM-001, 30 µM each) for 17 h. (**D**) HT22 cells were co-treated with DEP (100 µg/mL) and RSL3 (75 nM) in the absence or presence of EPAC2 regulators (inhibitor: ESI-05, 30 μM; activator: S220, 10 μM) for 17 h. (**E**) HT22 cells were co-treated with DEP (100 µg/mL) and RSL3 (75 nM) in the absence or presence of PDE4 inhibitor (rolipram, 30 μM) or PDE3 inhibitor (cilostamide, 30 μM) for 17 h. Cell metabolic activity was assessed by MTT assay. Data are presented as mean ± SD, each experiment containing 3 technical replicates. One-way ANOVA statistical analysis was used, *** *p* < 0.001, **** *p* < 0.0001. All experiments were independently repeated at least three times.

In a previous study from our group, we reported that pharmacological inhibition of EPAC1 prevented erastin-induced ferroptotic HT22 cell death [25]. To study the potential role of EPAC1 in RSL3-induced ferroptosis cell death, we used the pharmacological EPAC1 inhibitor CE3F4 in a concentration range of 3–100 µM. Concentrations ≥ 10 µM CE3F4 markedly prevented RSL3-induced cell death in HT22 cells (Figure S1B). Based on these results, we used 30 µM CE3F4 to further analyze the role of EPAC1 in DEP/RSL3-induced ferroptosis. First, Hoechst 33258/PI staining was carried out to visualize living and dead cells with different treatments, visualized at low (Figure S2A) and high (Figure 2A) magnification. As shown in Figure 2A, almost no red PI-positive cells were observed in the control or in cells treated with DEP alone. However, PI-positive cells significantly increased in the group co-treated with RSL3 and DEP compared to the group treated with RSL3 alone. CE3F4 reduced PI-positive cells, as shown by fewer red cells in the image. Quantification of PI-positive cells among total cells (relative to control) also showed that CE3F4 effectively blocked the ferroptotic cell death induced by DEP and RSL3 from 85.53 ± 6.77% to 35.81 ± 14.85% (Figure 2B). In addition to CE3F4, the EPAC1 inhibitor AM-001 (30 µM) also significantly decreased the cytotoxic effect induced by DEP and RSL3 co-treatment. As shown in Figure 2C, CE3F4 and AM-001 increased cell metabolic activity from 27.06 ± 11.14% to 87.56 ± 13.27% and 90.59 ± 1.84%, respectively. Cell fractionation followed by Western blot analysis demonstrated that EPAC1 is localized in both cytosol and mitochondria in HT22 cells [25]. As shown in Figure S2B, CE3F4 (30 µM) and AM-001 (30 µM) did not change the protein expression level of EPAC1.

The second messenger cAMP not only acts via EPAC1 but also via EPAC2 and PKA, and cellular gradients of cAMP are confined by PDEs. To test whether PKA, EPAC2, and PDEs may influence HT22 cell death, single or co-treatment of DEP and RSL3 in the absence or presence of Rp-8-CPT-cAMPs (PKA inhibitor), ESI-05 (EPAC2 inhibitor), S220 (EPAC2 activator), rolipram (PDE4 inhibitor), and cilostamide (PDE3 inhibitor) was used. Analysis of cell metabolic activity measurements showed that inhibition of PKA did not alter cell death induced by DEP and RSL3 (Figure S3C). Similar results were obtained with the EPAC2 inhibition and activation; the impaired cell metabolic activity caused by DEP and RSL3 exposure was not prevented by ESI-05 or S220 (Figure 2D). PDE inhibitors did not alter HT22 cell metabolic activity (Figure 2E). Collectively, our results implicated that DEP and RSL3 treatment triggered HT22 cell death in a process involving EPAC1, largely independent of EPAC2, PKA, or PDEs.

### 3.3. DEP and RSL3 Combined Exposure Increase Ca^2+^ and Induces sAC Activation

**Figure 3:**
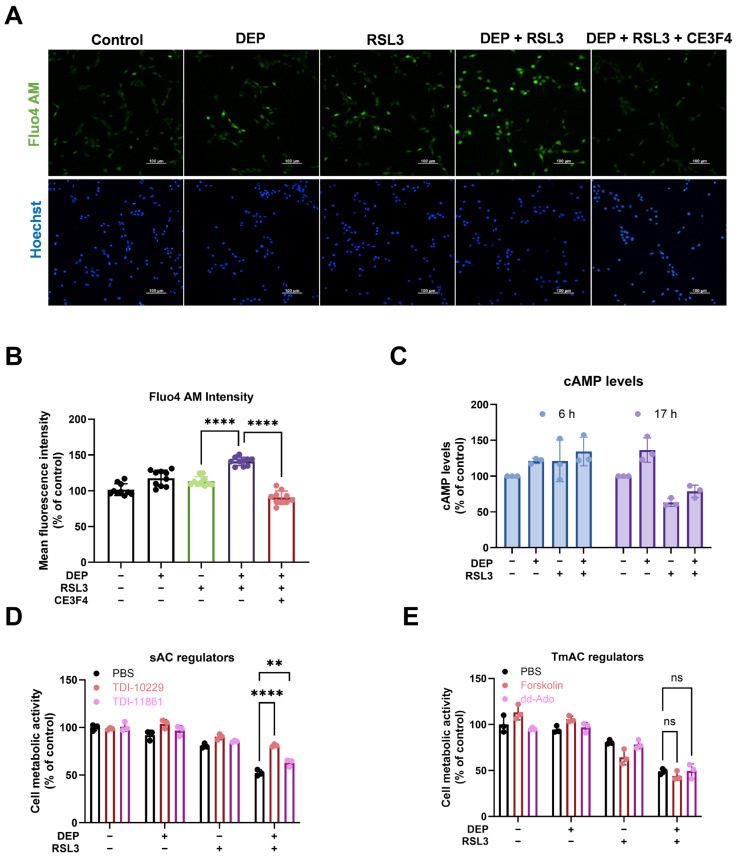
DEP and RSL3 co-exposure increase cytosolic Ca^2+^ and change cAMP levels in a process potentially involving sAC. (**A**) Representative pictures of fluorescent dye Fluo4-AM (green) in HT22 cells upon different treatments for 6 h. Scale bar = 100 µm. (**B**) Quantification of Fluo4 AM in HT22 cells following different treatment conditions. Data are presented as mean ± SD, with 10 randomly selected fields analyzed per condition in each experiment. (**C**) Intracellular cAMP levels measured by LC-MS/MS after 6 h and 17 h treatment (normalized to protein content). (**D**) HT22 cells were co-treated with DEP (100 µg/mL) and RSL3 (75 nM) in the absence or presence of sAC inhibitor (TDI-10229, 10 μM, and TDI-11861, 10 μM) for 17 h. (**E**) HT22 cells were co-treated with DEP (100 µg/mL) and RSL3 (75 nM) in the absence or presence of TmAC regulators (Forskolin, 30 μM, and dd-Ado, 10 μM) for 17 h. Cell metabolic activity was assessed by MTT assay. Data are presented as mean ± SD, each experiment containing 3 technical replicates. One-way ANOVA statistical analysis was used, ns *p* > 0.05, ** *p* < 0.01, **** *p* < 0.0001. All experiments were independently repeated at least three times.

Ca^2+^ homeostasis is crucial for various neuronal functions, but excessive Ca^2+^ is detrimental to neuronal function. Increased Ca^2+^ has been shown to be associated with ferroptosis [13,50,58]. As shown in Figure 3A,B, 100 μg/mL DEP alone slightly increased Ca^2+^ fluorescent intensities; however, it did not reach significant levels. Co-incubation with DEP and RSL3 increased cytosolic Ca^2+^ (141.15 ± 5.81%) compared to RSL3 treatment alone (113.47 ± 6.53%). Furthermore, treatment of HT22 cells with 30 μM CE3F4 reduced cytosolic Ca^2+^ levels (90.50 ± 9.23%), suggesting that EPAC1 inhibition maintained basal Ca^2+^ levels in the cytosol during ferroptotic cell death caused by DEP and RSL3 co-treatment.

As sAC can be activated by Ca^2+^ next to HCO_3_^−^ [58], we addressed whether DEP potentially further changes cAMP levels by the surrogate analyte-based LC-MS/MS method [49]. We noticed that combined exposure of DEP and RSL3 tended to further increase cAMP levels without any significant differences between each treatment group after 6 h (Figure 3C). However, cAMP production decreased after exposure to RSL3 alone or the combined treatment with NIST DEP and RSL3 compared to the control after 17 h, whereas the combined group showed higher cAMP levels compared to RSL3 treatment alone. As it is well established that activation of AC increases the intracellular level of cAMP [59], we investigated the possible involvement of TmAC and sAC using pharmacological activators/inhibitors in HT22 cells. Both sAC inhibitors, TDI-10229 and TDI-11861 (Figure 3D), significantly alleviated the decline of cell metabolic activity challenged by DEP and RSL3. In contrast, activation of TmAC, neither by forskolin nor by the p-site inhibitor dd-Ado, did not change the metabolic activity in HT22 cells challenged with DEP and RSL3 (Figure 3E). Forskolin increased the phosphorylation of PKA substrates, as shown in Figure S2D. To evaluate the localization of EPAC1 and sAC, immunofluorescence staining of HT22 cells was performed using fluorescence microscopy (Figure S3B,C). In summary, sAC and subsequent cAMP alterations seem to be involved in EPAC1-dependent ferroptosis following the stimulation with DEP and RSL3.

### 3.4. Combined Exposure of DEP and RSL3 Increases Mitochondrial ROS

**Figure 4:**
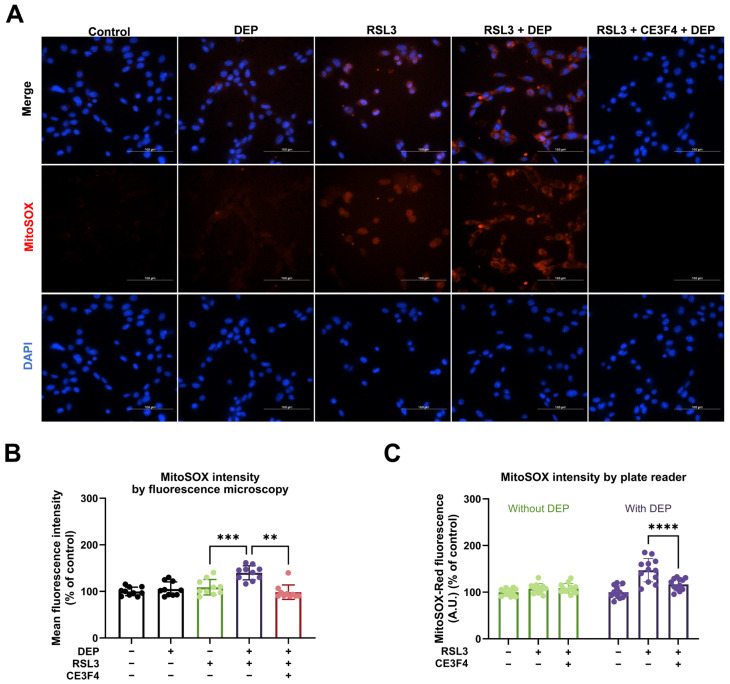
Mitochondrial ROS increase under the co-treatment with DEP and RSL3 in HT22 cells. (**A**) Representative pictures of MitoSOX (red) and nuclear Hoechst (blue) in HT22 cells. Scale bar = 100 µm. (**B**) Quantification of MitoSOX Red fluorescence intensity from imaging. Data are presented as mean ± SD, with at least 10 randomly selected fields analyzed per condition in each experiment. (**C**) MitoSOX Red fluorescence measured by plate reader. Data are presented as mean ± SD, with technical replicates averaged within each experiment. One-way ANOVA statistical analysis was used, ** *p* < 0.01, *** *p* < 0.001, **** *p* < 0.0001. All experiments were independently repeated at least three times.

Cellular ROS plays a vital part in HT22 cell death, which can be prevented by EPAC1 inhibition [25]. To determine whether DEP caused more cell death through mtROS, we used the MitoSOX red probe, a ROS superoxide indicator specifically targeting mitochondria in live cells. As shown in Figure 4A,B, mtROS production did not change in response to DEP alone. However, DEP reinforced the mtROS production under the challenge of RSL3. Additionally, increased mtROS induced by DEP and RSL3 co-treatment was reduced by the EPAC1 inhibitor, CE3F4. These results were confirmed by measurements of MitoSOX red fluorescence using a microplate reader (Figure 4C). After treatment with 75 nM RSL3, HT22 cells showed an increased mtROS level of 107.75 ± 10.30% compared to the control. Because the black color of DEP can affect fluorescence readings, the combined exposure group results were corrected with the DEP group. It was found that 100 μg/mL of DEP combined with 75 nM RSL3 exhibited higher mtROS levels of 147.14 ± 25.09% compared to the DEP group. CE3F4 could decrease mtROS levels (116.99 ± 12.59%) in HT22 cells caused by DEP and RSL3 co-treatment (Figure 4C). These results suggest that the mtROS decrease may be involved in the protective effect of EPAC1 pharmacological inhibition on DEP and RSL3-induced ferroptosis.

### 3.5. Combined Exposure to DEP and RSL3-Induced Lipid Peroxidation

**Figure 5:**
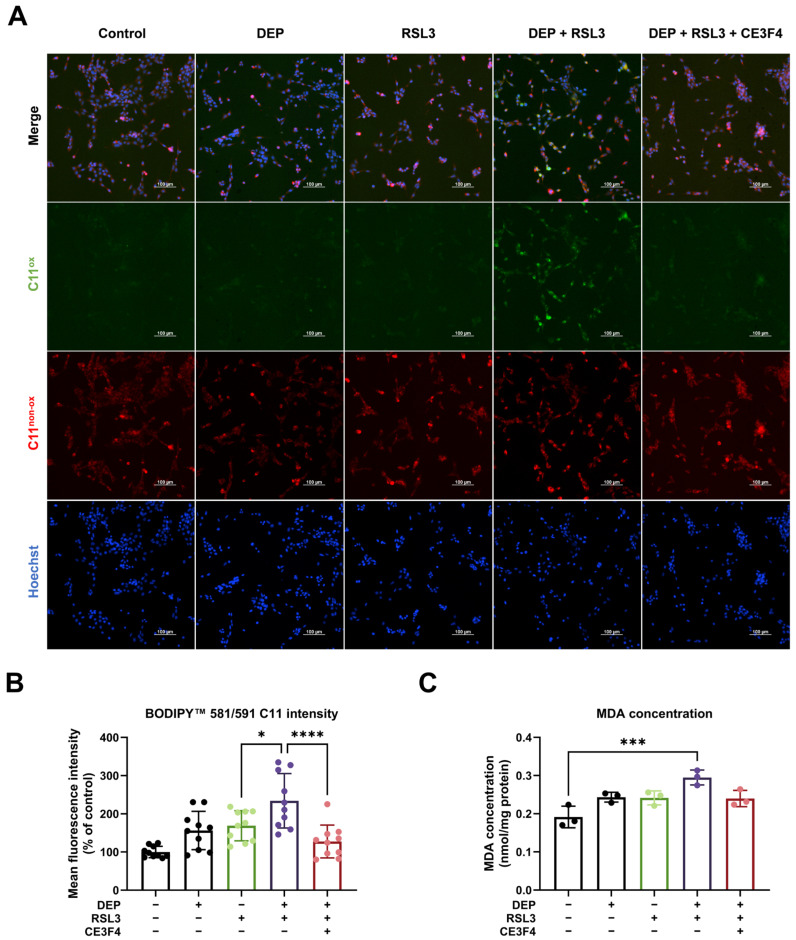
Lipid peroxidation increases in HT22 cells under the co-treatment with DEP and RSL3. (**A**) Representative images of BODIPY 581/591 C11 (red/green) and nuclear Hoechst (blue) in HT22 cells upon different treatments for 6 h. Scale bar = 100 µm. (**B**) Quantification of the green/red fluorescence ratio. Data are presented as mean ± SD, with at least 10 randomly selected fields analyzed per condition in each experiment. (**C**) MDA levels measured by TBARS assay after 6 h treatment (normalized to protein content). Data are presented as mean ± SD, normalized to protein concentration. One-way ANOVA statistical analysis was used, * *p* < 0.05, *** *p* < 0.001, **** *p* < 0.0001. All experiments were independently repeated at least three times.

Lipid peroxidation is another hallmark of ferroptosis [25]. To further investigate if lipid peroxidation contributes to increased neuronal cell death, we used BODIPY 581/591 C11, a sensitive fluorescent probe to monitor lipid peroxidation. The unoxidized molecule emits in the red spectrum, and the oxidized molecule emits in the green spectrum. As shown in Figure 5A, HT22 cells exposed to DEP exhibited a low ratio of oxidized (green) to non-oxidized (red) probe signal, whereas the combination of DEP and RSL3 showed higher green-to-red signal ratios compared to RSL3 treatment alone. CE3F4 decreased the green-to-red ratio in HT22 cells treated with DEP and RSL3. The results were further confirmed by quantification of green and red signal intensity (Figure 5B). MDA, one of the final products of polyunsaturated fatty acid peroxidation, was measured by the TBARS assay [54]. DEP in combination with RSL3 caused an increase in MDA concentration compared to RSL3 treatment (Figure 5C). CE3F4 decreased the MDA content caused by combined exposure, suggesting that EPAC1 is involved in the accumulation of oxidized lipids in HT22 cells with combined DEP and RSL3 exposure. Collectively, these data indicate that EPAC1 inhibition could rescue lipid peroxidation caused by DEP and RSL3.

## 4. Discussion

**Figure 6:**
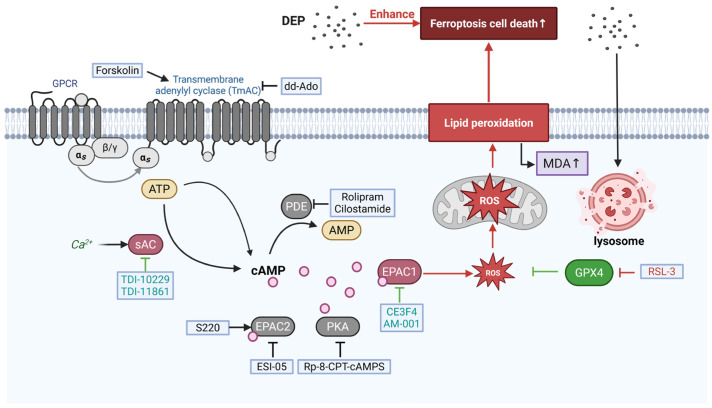
DEP sensitizes hippocampal neurons to ferroptosis by inducing lysosomal de-acidification and dysregulation of calcium homeostasis. This activates the sAC/cAMP/EPAC1 signaling axis, leading to increased mitochondrial ROS production and lipid peroxidation, ultimately accelerating ferroptotic cell death. Pharmacological inhibition of EPAC1 or sAC effectively blocks this pathway and provides neuroprotection. Key pharmacological interventions are shown: RSL3 (GPX4 inhibition), Fer-1 (ferroptosis inhibitor), QVD (caspase inhibitor), CE3F4 and AM-001 (EPAC1 inhibitors), ESI-05 (EPAC2 inhibitor), Sp-8-BnT-cAMPS (EPAC2 activator), rolipram and cilostamide (PDE inhibitors), TDI-10229 and TDI-11861 (sAC inhibitors), forskolin (tmAC activator), and dd-Ado (tmAC inhibitor). Created in BioRender. Menge, A. (2026) https://BioRender.com/rokupa0.

There is a growing interest in the possible role of PM in neurodegenerative diseases. PM seems to contribute to and exacerbate the onset of different neurodegenerative diseases due to the induction of oxidative stress, inflammation, and neuronal degeneration [60,61]. PM exposure has been found to cause neuronal hypocellularity and deficits in spatial memory in APP/PS1 mice [62]. In addition, PM exposure exacerbates motor impairment and dopaminergic neuronal death in 1-methyl-4-phenyl-1,2,3,6-tetrahydropyridine (MPTP)-induced PD mouse models [63]. The hippocampus plays a crucial role in cognition, learning, and memory processing and is more susceptible to oxidative stress caused by PM than other brain structures [64]. Dysregulated ferroptosis pathways are hypothesized to trigger neurodegeneration. However, the effects of air pollutants (e.g., DEP) on neurodegenerative diseases (ferroptotic hippocampal neurons) are still poorly understood, particularly regarding underlying potential mechanisms. In this study, we demonstrated that exposure to DEP enhanced RSL3-induced ferroptosis in HT22 cells and aggravated the activation of the sAC/cAMP/EPAC1 signaling pathway. Pharmacological inhibition of EPAC1 prevented the increased ferroptotic HT22 cell death and oxidative stress caused by DEP (Figure 6).

Our findings demonstrate that exposure to DEP at concentrations lower than 100 μg/mL did not significantly change HT22 cell metabolic activity, as measured by an MTT assay (Figure 1A). This observation is consistent with our previous reports [31,46] and also the study by Milani et al. [55], which reported that HT22 cell metabolic activity was not affected by 3–24 h of DEP exposure. Importantly, co-treatment with DEP (100 μg/mL) further increased RSL3-induced (50, 75 nM) cell death (Figure 1B), suggesting a synergistic effect between DEP and ferroptosis inducers. To elucidate the underlying mechanisms, we used the ferroptosis inhibitor ferrostatin-1 and the pan-caspase inhibitor QVD. Our results confirmed that ferroptosis, rather than apoptosis, is the primary mode of cell death accelerated by DEP (Figure 1C–E). These findings align with previous studies showing that ferroptosis is not consistently modulated by caspase inhibitors such as QVD [42] or Z-VAD [65]. The synergistic effect of DEP and RSL3 on ferroptosis highlights the potential role of environmental pollutants in exacerbating ferroptosis-related cell death. This is particularly relevant in the context of neurodegenerative diseases, where ferroptosis has been implicated as a key driver of neuronal loss.

We have shown before that EPAC1 is involved in ferroptotic cell death in HT22 cells [25]. In EPAC1-silenced HT22 cells, we observed no induction of ferroptosis. Here, we used CE3F4 (EPAC1 uncompetitive antagonist) and AM-001 (EPAC1 noncompetitive antagonist) as pharmacological tools to study EPAC1 signaling routes [66]. Our data show that CE3F4 and AM-001, both at 30 μM, significantly reduced DEP and RSL3-induced ferroptotic cell death (Figure 2C). This aligns with previous studies showing that EPAC1 regulates mitochondrial function and oxidative stress in other cell types [67,68]. Their distinct mechanisms of action and lack of effects in control cells suggest high specificity for EPAC1 without significant off-target effects. Interestingly, EPAC1 expression remained unchanged during ferroptosis (Figure S2A), suggesting that its activity, rather than abundance, drives cell death. This is consistent with the known role of EPAC1 as a cAMP-dependent signaling hub that modulates Ca^2+^ dynamics and mitochondrial function.

Cyclic AMP is degraded by PDE and is essential for the activation of EPAC and PKA. To elucidate the role of other cAMP transducers (PKA, EPAC2, and PDEs) besides EPAC1 in our cell system, specific inhibitors and activators have been utilized, such as Rp-8-CPT-cAPs, a PKA inhibitor, ESI-05, a selective inhibitor of EPAC2 [69], Sp-8-BnT-cAMPS (S220), a selective EPAC2 activator [70], rolipram, a selective inhibitor of PDE4 [71], and cilostamide, which is an inhibitor of PDE3 [72]. While EPAC1 plays a central role in DEP-induced ferroptosis, other cAMP signaling pathways (e.g., PKA, EPAC2, PDEs) appear to be less critical in our model. This contrasts with studies in other cell types, where PKA or EPAC2 activation has been linked to ferroptosis [73] or apoptosis [74]. These differences may reflect that cAMP, PKA, EPAC, and PDEs have differential roles in different systems, potentially depending upon stimuli, cell death pathways, and their relative abundance, distribution, localization, and specific cellular environments.

Given that RSL3 elevates intracellular Ca^2+^ [46,75], we hypothesized that DEP exacerbates ferroptosis by increasing Ca^2+^ levels. By using Fluo4 AM, we observed a significant increase in intracellular Ca^2+^ in cells treated with DEP and RSL3, which was attenuated by the EPAC1 inhibitor CE3F4 (Figure 3). Consistent with our data, Laudette et al. [68] reported that cAMP produced by sAC could activate EPAC1, leading to ROS accumulation in mitochondria. Additionally, environmental pollutants like Benzo(a)pyrene have been shown to increase Ca^2+^ through the sAC/cAMP/EPAC1/IP3 pathway, further supporting our observations [76]. The increase in intracellular Ca^2+^ during DEP and RSL3 co-treatment is likely due to release from intracellular stores, particularly the endoplasmic reticulum (ER) via inositol 1,4,5-trisphosphate receptors (IP3R) and mitochondria via voltage-dependent anion channel 1 (VDAC1), as suggested by our previous proximity ligation assay results [46]. DEP may exacerbate this process by inducing oxidative stress, leading to dysregulated calcium homeostasis. Inhibitors of the mitochondrial calcium uniporter (MCU), such as MCUi4 and RU265, attenuated cytosolic Ca^2+^ increases, supporting the role of mitochondrial calcium overload in ferroptosis. Since EPAC1 is activated by cAMP, we hypothesized that ferroptotic cell death induced by DEP and RSL3 might involve alterations in intracellular cAMP levels. Indeed, LC-MS/MS analysis revealed that combined treatment with DEP and RSL3 significantly increased cAMP levels compared to RSL3 alone (Figure 3C). This suggests that cAMP production is upstream of cytotoxicity and may play a role in promoting cell death. Increased intracellular cAMP levels can mediate both neuroprotective and cytotoxic effects in a context-dependent manner. While cAMP is often associated with neuronal differentiation and survival, its elevation can also disrupt astrocyte function and increase vulnerability to oxidative stress, ultimately promoting cell death pathways [77]. The increase in cAMP levels in our study underscores its potential as a critical signaling molecule in ferroptosis, particularly in response to combined environmental and pharmacological stressors.

The major source of cAMP is ATP converted by TmAC or sAC. As shown in this study, increased ferroptotic cell death induced by DEP was diminished in the presence of sAC inhibitor TDI-10229 but not in the presence of TmAC activators/inhibitors (Figure 3D,E), suggesting that DEP could increase the pool of cAMP via the sAC, but not TmAC. These findings highlight sAC as a critical mediator of cAMP-dependent ferroptosis in response to environmental stressors.

Long-term Ca^2+^ overload leads to increased ROS production, bioenergetic and metabolic disorders, and induction of cell death [13]. EPAC1 inhibition reduces ROS levels and lipid peroxidation by decreasing mitochondrial superoxide [25]. Activation of the ferroptosis pathway causes an elevation of oxidative stress, particularly in mitochondria, where mtROS contributes to lipid peroxidation of membranes. In our study, EPAC1 inhibition reduced mitochondrial superoxide levels (Figure 4) and lipid peroxidation (Figure 5), suggesting that EPAC1 activation exacerbates ferroptosis by promoting mitochondrial oxidative stress. This is consistent with previous findings that EPAC1 acts as a metabolic sensor, leading to lipid accumulation, mitochondrial dysfunction, and cardiomyocyte death [68]. In conclusion, EPAC1 inhibition confers protective roles in DEP-induced ferroptotic HT22 cells via inhibiting oxidative stress.

Oxidative stress is known to impair lysosomal function, particularly by inducing lysosomal de-acidification and membrane destabilization, which can lead to the release of lysosomal contents, including labile iron and hydrolases, thereby amplifying ROS production and lipid peroxidation [78,79]. The activation of lysosomal stress responses—a protective signaling cascade aimed at restoring lysosomal homeostasis, often involves transcription factor EB-mediated lysosomal biogenesis and autophagy [80]. In the context of air pollution, PM2.5 exposure has been shown to induce lysosomal stress in neuronal cells, contributing to ferroptosis sensitivity [41]. Importantly, our data indicate that DEP acts primarily as a sensitizing factor rather than as an independent ferroptotic trigger under the present experimental conditions. This may involve particle uptake and lysosomal trafficking, disturbed intracellular Ca^2+^ handling, and enhanced ER–mitochondria communication. In support of this interpretation, DEP-associated particles trafficked to lysosome-associated compartments were detected within HT22 cells (Figure S4), while DEP/RSL3 co-treatment increased ER–mitochondria contact sites and was functionally sensitive to perturbation of mitochondrial Ca^2+^ handling [46]. Because sAC is activated by intracellular Ca^2+^ and bicarbonate, DEP-induced Ca^2+^ dysregulation provides a biologically plausible route for enhanced cAMP generation and downstream EPAC1 signaling.

Our findings suggest that EPAC1-mediated ferroptosis may contribute to neuronal loss in neurodegenerative diseases such as AD and PD, where PM exposure exacerbates amyloid beta plaque accumulation and alpha-synuclein aggregation [11,12]. Recent work has also shown that PM2.5 can promote ferroptosis-associated inflammatory responses in microglia through Nrf2/Hmox1-related mechanisms [81]. At the same time, direct in vivo evidence linking air-pollution exposure specifically to EPAC1 activation in the brain remains limited. Therefore, our study identifies EPAC1 as a mechanistically supported mediator of mitochondrial ROS and calcium dynamics, positioning it as a potential therapeutic target. However, our in vitro model using HT22 cells may not fully recapitulate the complexity of in vivo systems. The brain response to particulate exposure is unlikely to be neuron-autonomous. Glial cells, particularly microglia and astrocytes, are likely to modulate DEP-induced neuronal vulnerability through neuroinflammatory signaling, ROS amplification, cytokine release, and neuron–glia crosstalk. Future studies should therefore extend these findings to primary neurons, neuron–glia co-cultures, organoid systems, and in vivo exposure models to validate the sAC/cAMP/EPAC1 axis and assess the therapeutic potential of EPAC1 inhibition in human-relevant conditions.

## 5. Conclusions

Our study demonstrates that DEP promotes RSL3-induced ferroptotic cell death in HT22 cells via the sAC/cAMP/EPAC1 axis, highlighting EPAC1 as a potential therapeutic target for neurodegenerative diseases exacerbated by air pollution. Future research should validate these findings in vivo models and explore pharmacological EPAC1 inhibitors as novel treatments for PM-related neuronal damage.

## Data Availability

The original contributions presented in this study are included in the article/Supplementary Material. Further inquiries can be directed to the corresponding author(s).

## Supplementary Materials

The following supporting information can be downloaded at https://www.mdpi.com/article/10.3390/antiox15050566/s1. Figure S1: Effects of cAMP signalling modulators on DEP/RSL3-induced cytotoxicity in HT22 cells; (A) HT22 cells were treated with RSL3 at the indicated concentrations for 17 h. (B) HT22 cells were treated with indicated concentrations of combinations of CE3F4 and RSL3 for 17 h. (C) HT22 cells were cotreated with NIST DEP (100 µg/mL) and RSL3 (75 nM) in the absence or presence of PDE4 inhibitor (rolipram, 30 μM) for 17 h. (D) HT22 cells were cotreated with NIST DEP (100 µg/mL) and RSL3 (75 nM) in the absence or presence of PDE3 inhibitor (cilostamide, 30 μM) ) for 17 h. (E) HT22 cells were cotreated with NIST DEP (100 µg/mL) and RSL3 (75 nM) in the absence or presence of EPAC2 inhibitor (ESI-05, 30 μM) for 17 h. (F) HT22 cells were cotreated with NIST DEP (100 µg/mL) and RSL3 (75 nM) in the absence or presence of EPAC2 activator (S220, 10 μM) for 17 h. (G,H) HT22 cells were cotreated with NIST DEP (100 µg/mL) and RSL3 (75 nM) in the absence or presence of sAC inhibitor (TDI-10229, 10 μM and TDI-11861, 10 μM) for 17 h. MTT assay was performed, and data are presented as mean ± SD, n = 3–6, *** *p* < 0.001, **** *p* < 0.0001. Figure S2: Effects of EPAC1 inhibition on DEP/RSL3-induced cell death and EPAC1 protein levels in HT22 cells; (A) Fluorescence images of HT22 cells at 10X magnification after being cotreated with NIST DEP (100 µg/mL) and RSL3 (75 nM) in the absence or presence of EPAC1 inhibitor (CE3F4, 30 µM) for 17 h via PI and Hoechst 33452 staining (red, dead cells; blue, nuclei). Scale bar = 100 µm. (B) EPAC1 protein levels after NIST DEP/RSL3 treatment in the presence or absence of EPAC1 inhibitors. Figure S3: Validation of pharmacological inhibitors and activators in HT22 cells; (A) Schematic diagram displayed the simplified activation of PKA and EPAC proteins by cAMP. (B) HT22 cells were cotreated with NIST DEP (100 µg/mL) and RSL3 (75 nM) in the absence or presence of PKA inhibitor (Rp-8-CPT-cAMP, 100 μM) for 17 h. MTT assay was performed, and data are presented as mean ± SD, n = 3, *** *p* < 0.001, **** *p* < 0.0001. (C,D) Phospho-PKA substrates after Forskolin, Rolipram and Cilostamide treatment in HT22 cell. (E) Immunofluorescence staining of EPAC1 and sAC in HT22 cells by fluorescence microscopy. Scale bar = 20 µm. Figure S4: Internalization of DEP and DEP-induced lysosomal de-acidification in HT22 cells; (A,B) DEP internalization and lysosomal localization. HT22 cells were treated with 10 µg/mL DEP for 24 h, subsequently stained with LysoTracker red, and imaged at 60× magnification. (A) Fluorescence and brightfield mages showing DEP internalization. Dark spots are visible in both treated and untreated cells (green circles). However, (some of) the dark spots in the treated cells give a different appearance, having rougher edges, compared to the dark spots in untreated cells. Furthermore, (some of) the dark spots in treated cells also appear dark in the LysoTracker red channel, while such objects are not observed in treated cells. This shows that (some of) the dark spots in treated cells are DEP and consequently that DEP are internalized by cells. Scale bar = 5 or 20 µm. (B) z stack images demonstrating co-localization of DEP with lysosomes (green circles). Scale bar = 5 µm. (C) Representative phase-contrast and red fluorescence images showing lysosomal de-acidification over time. HT22 cells were stained with LysoView 540 and imaged at 0, 1, 2, and 17 h. Scale bar = 400 µm. (D) Quantification of LysoView 540 red fluorescence intensity in HT22 cells treated with DEP (30 or 100 µg/mL), RSL3 (75 nM), or their combination in the absence or presence of CE3F4 (30 µM), measured by Incucyte live-cell imaging system. (E) Quantification of LysoTracker Green fluorescence intensity by flow cytometry in HT22 cells treated with DEP alone at increasing concentrations (3, 10, 30, and 100 µg/mL) for 24 h. The results of 3 replicate samples are shown together with their average indicated by a line. (F) Quantification of LysoTracker Green fluorescence intensity by flow cytometry in HT22 cells treated with DEP (100 µg/mL), RSL3 (75 nM), or their combination in the absence or presence of EGTA (1 µM) or CE3F4 (30 µM). The results of 3 replicate experiments, each with 3 replicate samples are shown. Figure S5: Effect of Tween 80 on cell viability and membrane integrity. (A) Cells were treated with Medium, Tween 80 (0.1% *v*/*v*), and DEP. Cell viability was assessed, showing no significant difference between Tween 80–treated and untreated cells. (B) Representative fluorescence images of Hoechst/PI double staining after Tween 80 (0.1% *v*/*v*) treatment. Hoechst stains nuclei (blue), and PI labels dead cells (red).

## Abbreviations

The following abbreviations are used in this manuscript:

AC	adenylyl cyclase
AD	Alzheimer’s disease
ATP	adenosine triphosphate
Ca^2+^	calcium ion
cAMP	cyclic adenosine monophosphate
CNS	central nervous system
Cys	cysteine
DEP	diesel exhaust particles
EPAC	exchange protein directly activated by cAMP
Glu	glutamate
GPX4	glutathione peroxidase 4
GSH	glutathione
HBSS	Hanks’ balanced salt solution
HCO_3_^−^	bicarbonate
LC-MS/MS	liquid chromatography with tandem mass spectrometry
MDA	malondialdehyde
mtROS	mitochondrial ROS
MTT	the 3-[4,5-dimethylthiazol-2-yl]-2,5-diphenyl-2H-tetrazolium bromide
NIST	National Institute of Standards and Technology
PD	Parkinson’s disease
PDEs	phosphodiesterases
PKA	protein kinase A
PM	particulate matter
ROS	reactive oxygen species
RT	room temperature
sAC	soluble adenylyl cyclase
SRM	standard reference material
TBARS	thiobarbituric acid reactive substances
TmAC	transmembrane adenylyl cyclases
WHO	World Health Organization
