# Supplementary material for: Inhibition of EPAC1 Prevents Neuronal Death Mediated by Diesel Exhaust Particles in Ferroptotic Cell Death Conditions
Source: Antioxidants (Basel). 2026 Apr 29;15(5):566. doi: 10.3390/antiox15050566 (PMC13203856; doi:10.3390/antiox15050566)
Supplement: Supplementary file 1 [file antioxidants-15-00566-s001.zip › antioxidants-4257238-supplementary.pdf]

Inhibition of EPAC1 prevents neuronal death mediated by diesel exhaust particles in ferroptotic cell death conditions

Hong Yan [1], Leshan Zhang [1], Ana L Manzano-Covarrubias [1,2], Phoeja S Gadjdjoe [1], Anja Land [1], Christina H J T Mol van der Veen [1], Teresa Mitchell-Garcia[1], Heba A Fayyaz[3], Marco Venema[4], Christoffer Åberg[4], Marieke van der Hart [3,4], Frank Lezoualch [5], Xiaodong Cheng [6], Amalia M Dolga [1,*], Martina Schmidt [1,2,*]

[1] Department of Molecular Pharmacology, Groningen Research Institute of Pharmacy (GRIP), Faculty of Science and Engineering, University of Groningen, Antonius Deusinglaan 1, 9713 AV Groningen, The Netherlands
[2] Groningen Research Institute of Asthma and COPD (GRIAC), Groningen Research Institute of Pharmacy (GRIP), University Medical Center Groningen (UMCG), University of Groningen, 9713 AV Groningen, The Netherlands
[3] Department of Nanomedicine and Drug Targeting, Groningen Research Institute of Pharmacy, Faculty of Science and Engineering, University of Groningen, Antonius Deusinglaan 1, 9713 AV Groningen, The Netherlands
[4] Department of Pharmaceutical Analysis, Groningen Research Institute of Pharmacy, Faculty of Science and Engineering, University of Groningen, Antonius Deusinglaan 1, 9713 AV Groningen, The Netherlands
[5] Analytical Biochemistry, Groningen Research Institute of Pharmacy (GRIP), Faculty of Science and Engineering, University of Groningen, Antonius Deusinglaan 1, 9713 AV Groningen, The Netherlands
[6] Quantall B.V., L.J. Zielstraweg 1, 9713 GX Groningen, The Netherlands;
[7] Inserm UMR-1297, Institut des Maladies Métaboliques et Cardiovasculaires, Université Toulouse Paul Sabatier, 31400 Toulouse, France
[8] Department of Integrative Biology & Pharmacology, Texas Therapeutics Institute, University of Texas Health Science Center at Houston, Houston, TX 7000, USA
* Correspondence: m.schmidt@rug.nl (M. Schmidt) orcid.org/0000-0003-3075-0630; a.m.dolga@rug.nl (A.M. Dolga)

Supplementary figure 1

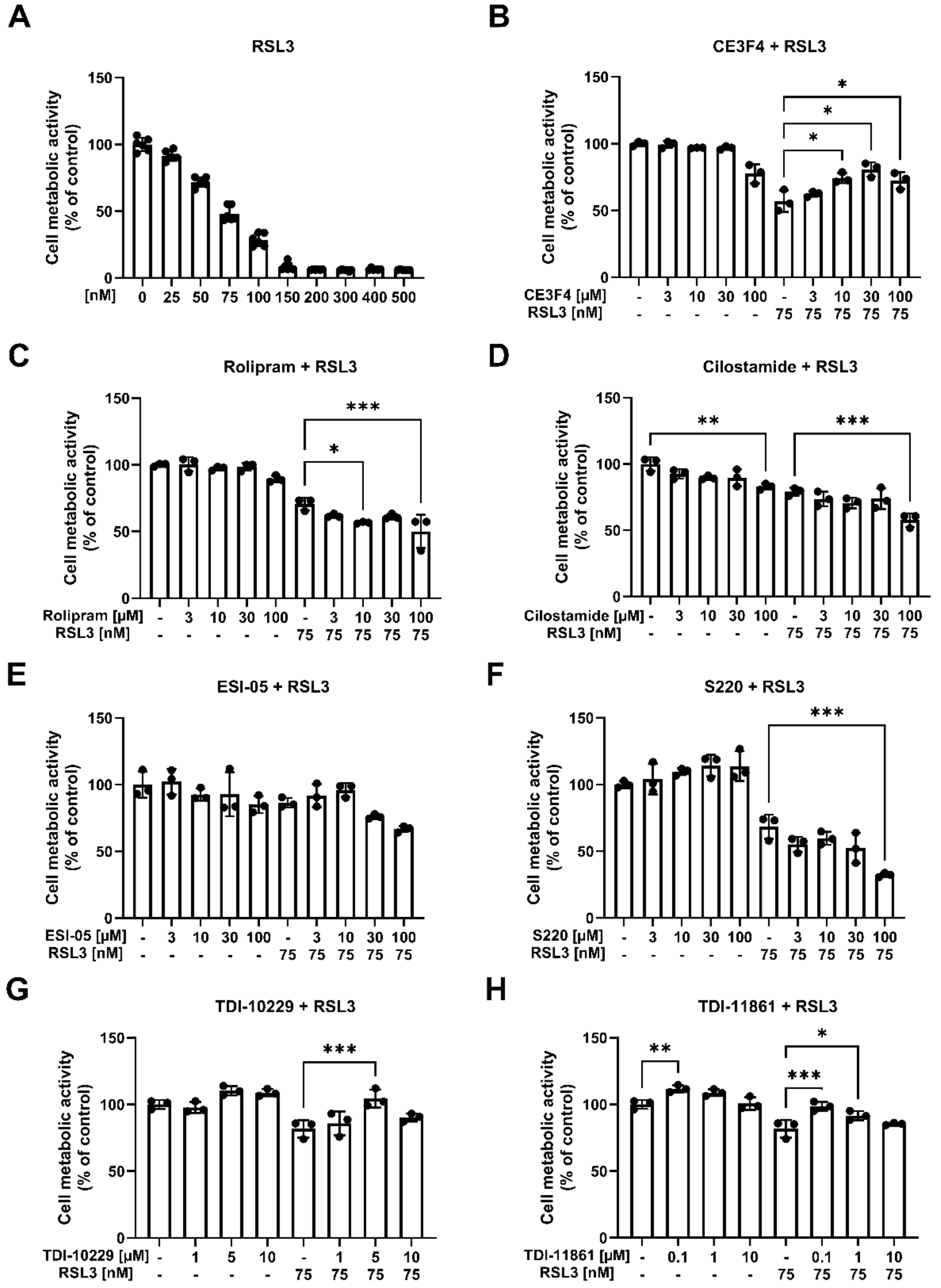

Supplementary Fig. 1. (A) HT22 cells were treated with RSL3 at the indicated concentrations for 17 h. (B) HT22 cells were treated with indicated concentrations of combinations of CE3F4 and RSL3 for 17 h. (C) HT22 cells were cotreated with NIST DEP (100 μg/mL) and RSL3 (75 nM) in the absence or presence of PDE4 inhibitor (rolipram, 30 μM) for 17 h. (D) HT22 cells were cotreated with NIST DEP (100 μg/mL) and RSL3 (75 nM) in the absence or presence of PDE3 inhibitor (cilostamide, 30 μM)) for 17 h. (E) HT22 cells were cotreated with NIST DEP (100 μg/mL) and RSL3 (75 nM) in the absence or presence of EPAC2 inhibitor (ESI-05, 30 μM) for 17 h. (F) HT22 cells were cotreated with NIST DEP (100 μg/mL) and RSL3 (75 nM) in the

absence or presence of EPAC2 activator (S220, 10 μM) for 17 h. (G,H) HT22 cells were cotreated with NIST DEP (100 μg/mL) and RSL3 (75 nM) in the absence or presence of sAC inhibitor (TDI-10229, 10 μM and TDI-11861, 10 μM) for 17 h. MTT assay was performed, and data are presented as mean ± SD, n=3-6, $^{***}p < 0.001$, $^{****}p < 0.0001$.

Supplementary figure 2

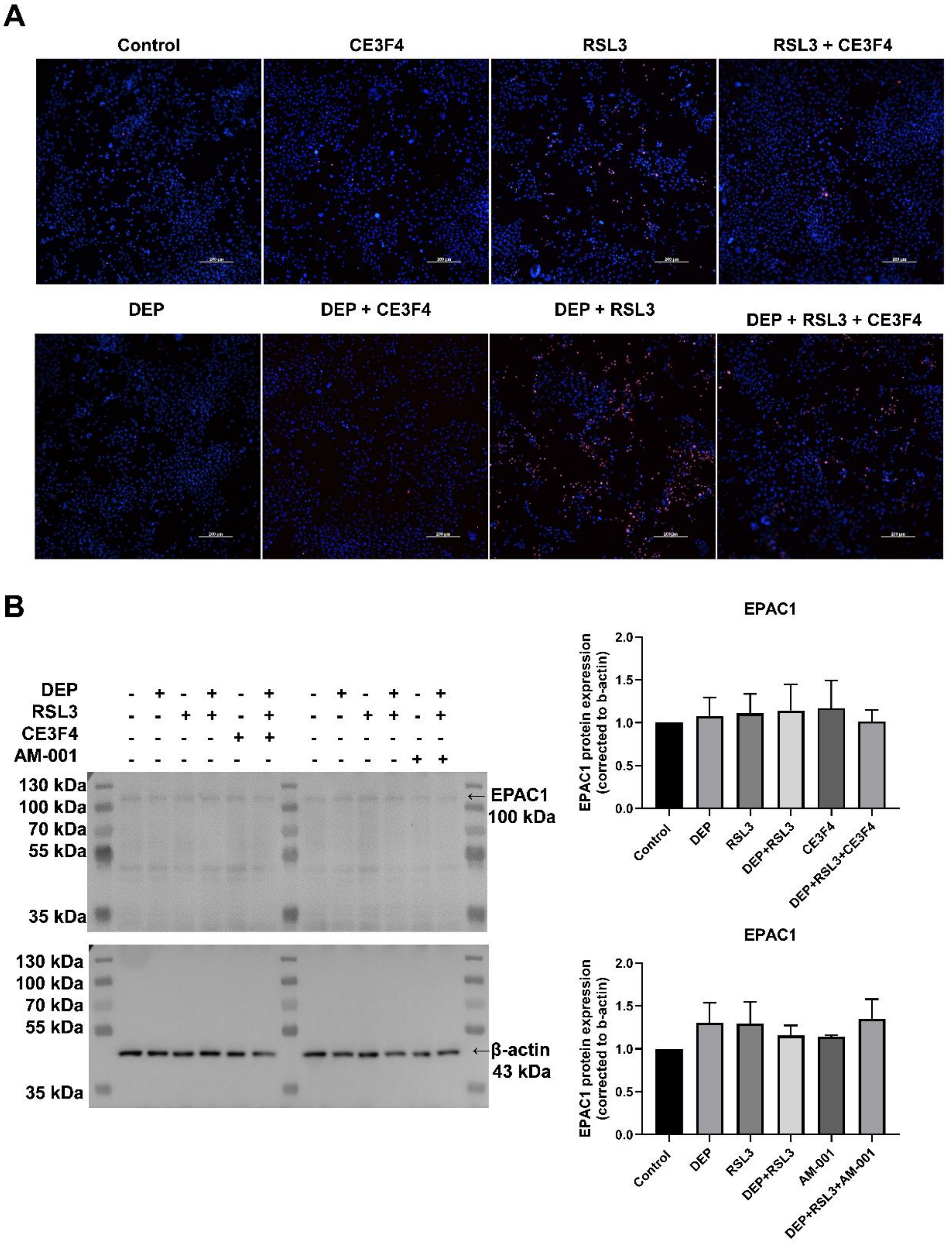

Supplementary Fig. 2. (A) Fluorescence images of HT22 cells at 10X magnification after being cotreated with NIST DEP (100 μg/mL) and RSL3 (75 nM) in the absence or presence of EPAC1 inhibitor (CE3F4, 30 μM) for 17 h via PI and Hoechst 33452 staining (red, dead cells; blue, nuclei). Scale bar = 100 μm. (B) EPAC1 protein levels after NIST DEP/RSL3 treatment in the presence or absence of EPAC1 inhibitors.

Supplementary figure 3

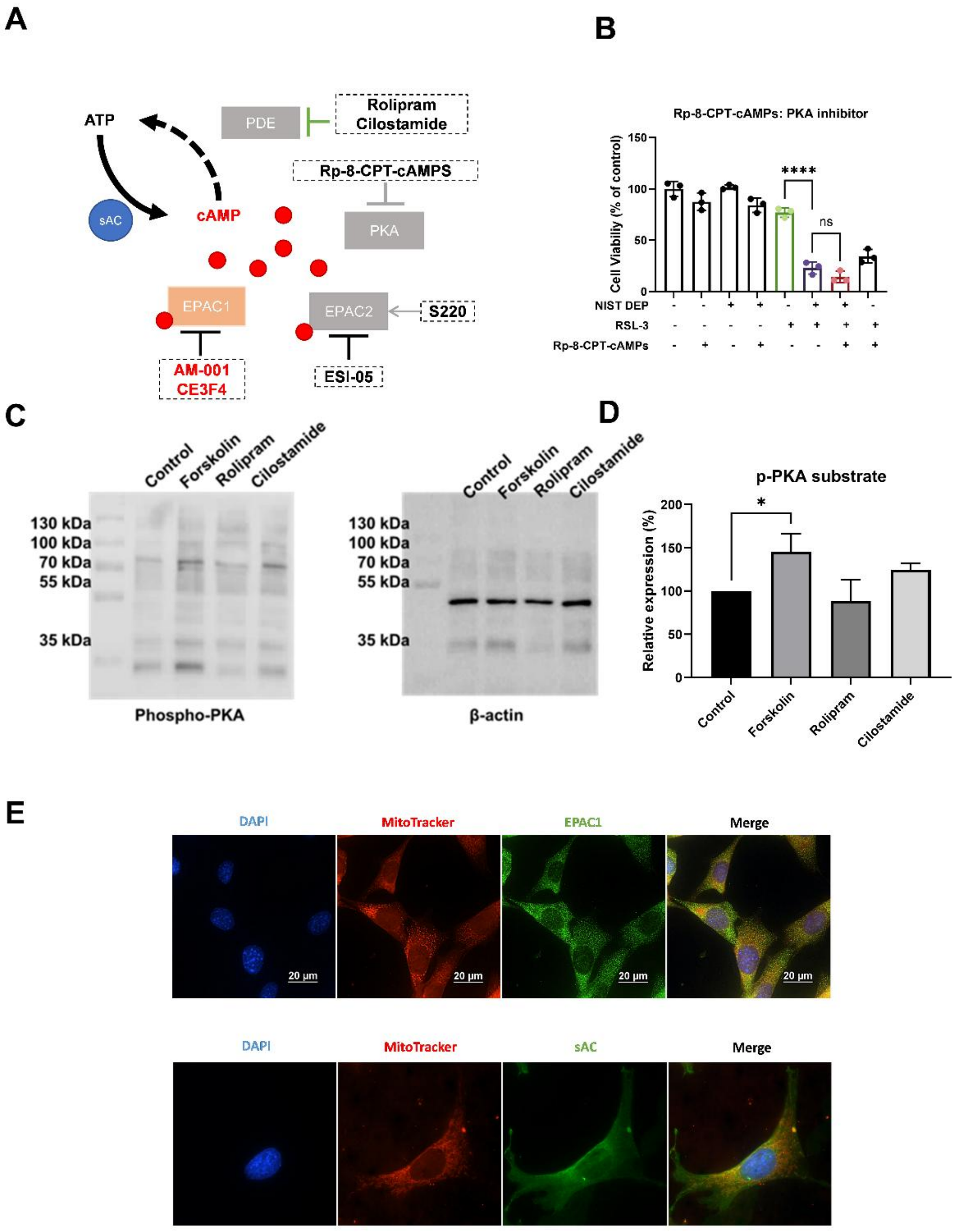

Supplementary Fig. 3. (A) Schematic diagram displayed the simplified activation of PKA and EPAC proteins by cAMP. (B) HT22 cells were cotreated with NIST DEP (100 μg/mL) and RSL3 (75 nM) in the absence or presence of PKA inhibitor (Rp-8-CPT-cAMP, 100 μM) for 17 h. MTT assay was performed, and data are presented as mean ± SD, n=3, $^{***}p < 0.001$, $^{****}p < 0.0001$. (C,D) Phospho-PKA substrates after Forskolin, Rolipram and Cilostamide treatment in HT22 cell. (E) Immunofluorescence staining of EPAC1 and sAC in HT22 cells by fluorescence microscopy. Scale bar = 20 μm.

Supplementary figure 4

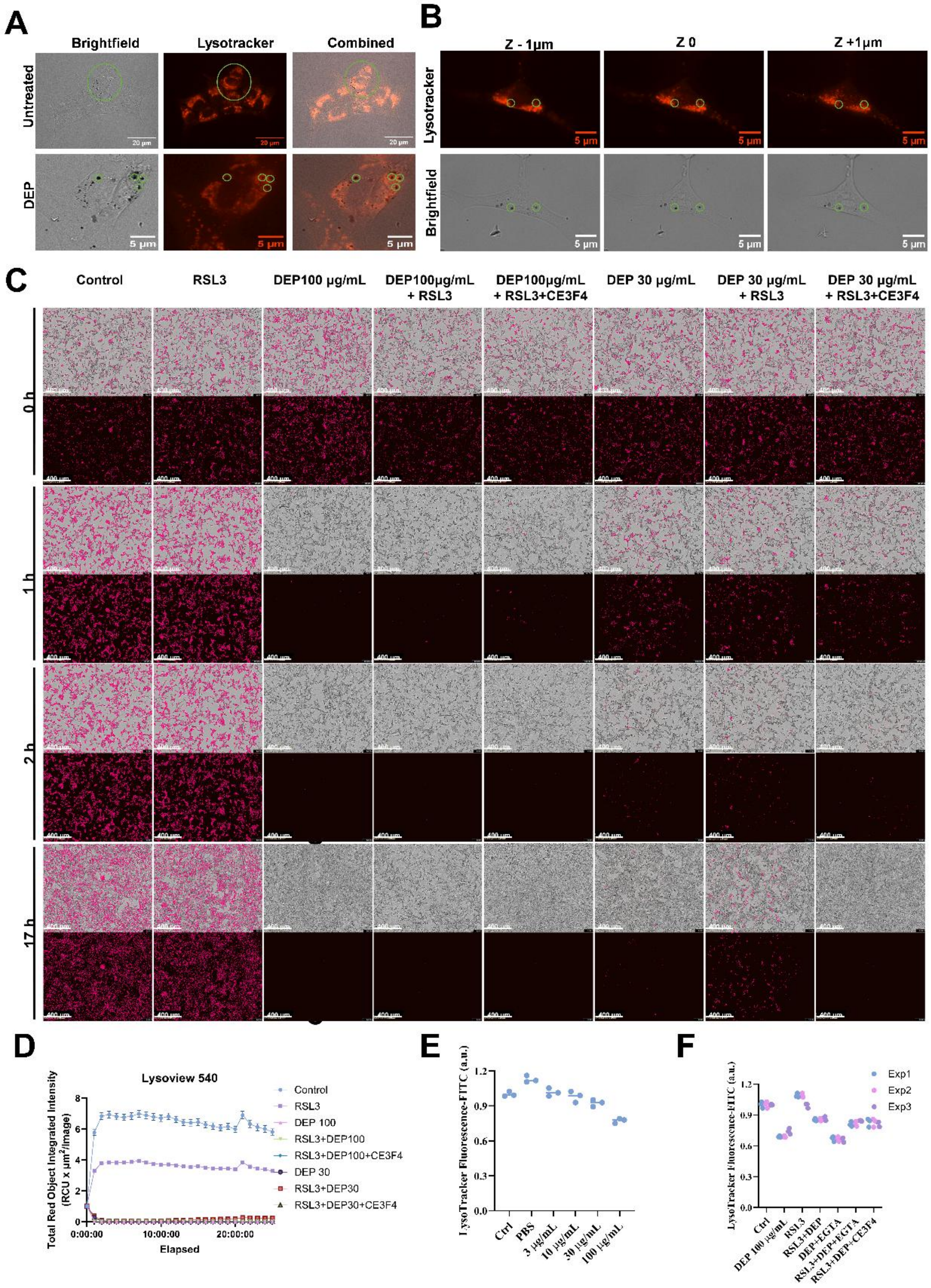

Supplementary Fig. 4. Internalization of DEP and DEP-induced lysosomal de-acidification in HT22 cells. (A-B) DEP internalization and lysosomal localization. HT22 cells were treated with 10 μg/mL DEP for 24 h, subsequently stained with LysoTracker red, and imaged at 60× magnification. (A) Fluorescence and brightfield mages showing DEP internalization. Dark spots are visible in both treated and untreated cells (green circles). However, (some of) the dark spots

in the treated cells give a different appearance, having rougher edges, compared to the dark spots in untreated cells. Furthermore, (some of) the dark spots in treated cells also appear dark in the LysoTracker red channel, while such objects are not observed in treated cells. This shows that (some of) the dark spots in treated cells are DEP and consequently that DEP are internalized by cells. Scale bar = 5 or 20 μm. (B) *z* stack images demonstrating co-localization of DEP with lysosomes (green circles). Scale bar = 5 μm. (C) Representative phase-contrast and red fluorescence images showing lysosomal de-acidification over time. HT22 cells were stained with LysoView 540 and imaged at 0, 1, 2, and 17 h. Scale bar = 400 μm. (D) Quantification of LysoView 540 red fluorescence intensity in HT22 cells treated with DEP (30 or 100 μg/mL), RSL3 (75 nM), or their combination in the absence or presence of CE3F4 (30 μM), measured by Incucyte live-cell imaging system. (E) Quantification of LysoTracker Green fluorescence intensity by flow cytometry in HT22 cells treated with DEP alone at increasing concentrations (3, 10, 30, and 100 μg/mL) for 24 h. The results of 3 replicate samples are shown together with their average indicated by a line. (F) Quantification of LysoTracker Green fluorescence intensity by flow cytometry in HT22 cells treated with DEP (100 μg/mL), RSL3 (75 nM), or their combination in the absence or presence of EGTA (1 μM) or CE3F4 (30 μM). The results of 3 replicate experiments, each with 3 replicate samples are shown.

Supplementary figure 5

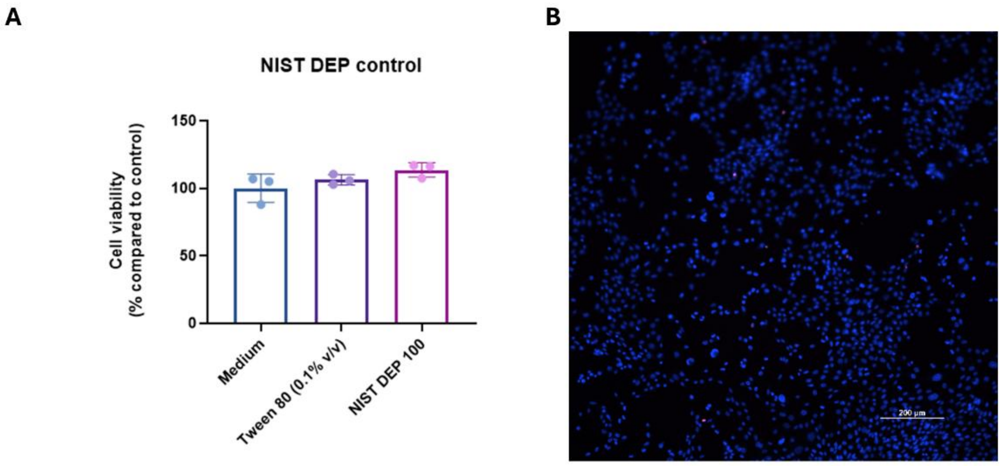

Supplementary Fig. 5. Effect of Tween 80 on cell viability and membrane integrity. (A) Cells were treated with Medium, Tween 80 (0.1% v/v), and DEP. Cell viability was assessed, showing no significant difference between Tween 80–treated and untreated cells. (B) Representative fluorescence images of Hoechst/PI double staining after Tween 80 (0.1% v/v) treatment. Hoechst stains nuclei (blue), and PI labels dead cells (red).
